# Stratifying cardiometabolic risk in urban Chinese children with obesity: Study protocol of the Shenzhen Children Cohort Study (SCCS)

**DOI:** 10.1371/journal.pone.0340049

**Published:** 2026-01-22

**Authors:** Yuehao Xu, Xin Guo, Jinwen Liao, Xiaolin Sun, Zhangcong Liang, Mingguo Xu

**Affiliations:** 1 Department of Pediatrics, The Third People’s Hospital of Longgang, Clinical Institute of Shantou University Medical College, Shenzhen, China; 2 Guangdong Key Laboratory for Biomedical Measurements and Ultrasound Imaging, National-Regional Key Technology Engineering Laboratory for Medical Ultrasound, School of Biomedical Engineering, Shenzhen University Medical School, Shenzhen, China; 3 Department of Experiment & Research, South China Hospital, Medical School, Shenzhen University, Shenzhen, China; 4 Department of Pediatrics, Longgang District Maternity & Child Healthcare Hospital of Shenzhen City (Longgang Maternity and Child Institute of Shantou University Medical College), Shenzhen, China; Longgang Otorhinolaryngology Hospital & Shenzhen Key Laboratory of Otorhinolaryngology, Shenzhen Institute of Otorhinolaryngology, CHINA

## Abstract

**Background:**

Cardiovascular risk factors often begin in early childhood, with obesity being a major contributor. However, not all children with obesity share the same risk profile. The Shenzhen Children Cohort Study (SCCS) is a prospective, multimodal cohort designed to follow overweight and obese children aged 5–11 years, aiming to identify subgroups with elevated long-term cardiometabolic risk. The study will longitudinally track the evolution of cardiometabolic risk by integrating anthropometric, biochemical, imaging, behavioral, and psychosocial data. In addition to profiling child-level risk trajectories, SCCS will examine how parental beliefs, health behaviors, and family environments shape the development and progression of obesity-related cardiovascular risks. By capturing interactions between family-level determinants and biological markers, the study aims to support individualized risk stratification and inform early-life prevention frameworks in urban China.

**Methods:**

The Shenzhen Children Cohort Study (SCCS) will enroll 3,363 overweight and obese children aged 5–11 years from Longgang District, Shenzhen, through public recruitment campaigns. Participants will undergo annual follow-up visits over a five-year period. At each visit, standardized clinical examinations, laboratory tests, and caregiver-completed questionnaires will be conducted to assess cardiovascular, behavioral, and environmental risk factors. All data will be centrally managed and analyzed using longitudinal statistical models to characterize cardiometabolic risk trajectories and to evaluate how family-level factors interact with child health indicators over time.

**Discussion:**

This study follows overweight and obese children aged 5–11 years over five years to document changes in physical measurements, biochemical indicators, imaging results, and questionnaire data on household structure, parental health history, and child routines. The aim is to build a multi-domain risk profile that moves beyond body mass index and simple metabolic categories. Prior studies often grouped children by BMI percentile or defined metabolically healthy and unhealthy types based on a few biomarkers. These methods overlook transitions, internal variation, and context. By applying repeated measurements and multiple modeling approaches, this study aim to identify subgroups based on shared risk trajectories, biomarker shifts, and family conditions. Risk is not treated as a single threshold but as a process shaped by exposures and responses. Although this cohort is located in one district, its structure may guide future work on pediatric risk classification under precision medicine frameworks.

## Introduction

Cardiovascular disease (CVD) remains one of the leading causes of death and a major source of healthcare burden globally [1]. Beyond its direct impact, CVD is closely associated with the onset and progression of numerous other diseases, further compounding its health and economic challenges [2–5]. While CVD is often diagnosed in adulthood, the risk factors contributing to its development often begin in childhood and adolescence. The accumulation of cardiovascular risk factors in early life, such as obesity, dyslipidemia, hypertension, congenital heart defects and coronary artery abnormalities, strongly influences long-term cardiovascular health. These conditions exert both immediate effects and lasting biological impacts. Evidence from cohort studies shows that children carrying multiple risk factors can develop early atherosclerotic changes and vascular dysfunction by adolescence. This progression underscores the growing recognition that early identification of risk is essential. As a result, early intervention has become a central strategy to slow the development of cardiovascular disease and reduce its long-term burden [6–10]. Among these early risk factors, obesity is both common and modifiable. However, not all children with obesity share the same future risk. Differences in biological markers, behavior, and family environment may lead to distinct health outcomes. Understanding this variation is essential for identifying which children require more intensive monitoring or intervention.

Early intervention targeting multiple cardiovascular risk factors requires comprehensive prevention strategies. A variety of evidence-based approaches have been investigated, including dietary modifications [11], structured physical activity programs, promotion of healthier lifestyles, innovative approaches like cell-based therapy for cardiovascular repair [12], and minimizing exposure to environmental risk factors such as tobacco smoke [6]. Dietary interventions that focus on reducing caloric intake and improving nutritional quality have been shown to improve weight management and metabolic health [13–16], while physical activity initiatives incorporating both aerobic and resistance training have shown effectiveness in reducing cardiometabolic risk factors [17,18]. Public health efforts also emphasize the importance of creating a supportive environment for children to adopt sustainable healthy behaviors [19]. Although these interventions perform well in controlled settings, their real-world effectiveness remains limited, especially in fast-changing urban environments such as Shenzhen.

Recent evidence shows that childhood cardiovascular risk does not follow a single pathway but emerges through different developmental patterns. This shift has moved research away from uniform approaches toward precision strategies that reflect individual differences in genetics, environment, and lifestyle. This paradigm shift has led to the recognition that cardiovascular risk develops through multiple pathways, requiring targeted intervention approaches [20]. Recent multi-omics approaches have enhanced our understanding of cardiometabolic disorders, revealing distinct molecular signatures and pathways that contribute to cardiovascular disease risk through various mechanisms [21]. These insights point to the importance of integrating biological measures with family-level factors when developing early risk identification strategies for children with obesity.

Several notable cohort studies worldwide have investigated cardiovascular risk factors in children and adolescents. The Bogalusa Heart Study pioneered the understanding of cardiovascular risk factor trajectories from childhood to adulthood, demonstrating that atherosclerotic changes begin in youth [22,23]. The Young Finns Study established the relationship between childhood lifestyle factors and adult cardiovascular outcomes, following over 3,500 children [24]. More recently, the Generation R Study (Netherlands) [25] and the QUebec Adipose and Lifestyle Investigation in Youth (QUALITY) (Canada) [26] have focused on genetic and environmental determinants of cardiovascular health in contemporary youth populations. In Asia, the Hong Kong Children of May 1997 Birth Cohort [27] showed unique patterns of cardiovascular risk factor clustering in Chinese children. Within mainland China, existing cohorts include the Beijing Child and Adolescent Metabolic Syndrome (BCAMS) study [28], which followed 19,593 children since 2004. However, the Chinese cohort primarily concentrated on metabolic syndrome or obesity in isolation, rather than comprehensive cardiovascular risk assessment. Moreover, none have specifically addressed the unique characteristics of rapid urbanization zones like Shenzhen, where lifestyle transitions occur at unprecedented rates [29]. The existing cohorts also predate significant technological and social changes that have dramatically altered children’s living environments in Chinese mega-cities over the past decade.

Shenzhen, as China’s first special economic zone and one of the fastest-growing metropolitan areas globally, presents both unique challenges and opportunities for studying childhood cardiovascular risk factors. The city’s rapid urbanization has brought significant changes in lifestyle, environmental exposures, and healthcare access that may impact children’s cardiovascular health. However, despite these rapid changes, there is a critical lack of longitudinal data on the prevalence and determinants of cardiovascular risk factors among children in urban Chinese settings like Shenzhen. This knowledge gap limits the understanding of how obesity-related cardiovascular risks develop during childhood, especially in rapidly urbanizing regions of China. To address this, the Shenzhen Children Cohort Study (SCCS) is designed as a longitudinal study focusing on overweight and obese children aged 5–11 years in Shenzhen, Guangdong Province. The study will collect detailed anthropometric, biochemical, behavioral, and family-level information to examine how biological and social factors jointly influence the progression of cardiometabolic risk. By following participants over time, SCCS will provide evidence on how individual and household characteristics shape early cardiovascular health trajectories. The findings are expected to inform risk prediction and stratification models that support personalized prevention in urban pediatric populations.

## Materials and methods

### Study design and setting

This study is a prospective cohort study targeting children aged 5–11 years who are classified as overweight or obese according to the Chinese national standard WS/T 586–2018 Screening for overweight and obesity in school-age children and adolescents, using age- and sex-specific BMI-for-age Z-score criteria. It is designed to investigate cardiometabolic risk trajectories and their associations with family-level factors. The study will be implemented at the Third People’s Hospital of Longgang. Participant recruitment will begin in March 1st 2026 and is expected to be completed by May 1st 2026. Each participant will undergo an annual follow-up visit conducted between March and May of every study year from 2026 to 2030. The five annual visits will include standardized physical examinations, laboratory testing, imaging assessments, and caregiver-completed questionnaires. This duration was selected to capture key developmental changes in cardiometabolic risk during late childhood while maintaining operational feasibility in a large urban population. The study is structured to allow extension into later phases as participants enter adolescence and adulthood, and insights from the initial phase will guide adjustments to measurement domains and follow-up intervals in subsequent stages. Data collection is expected to conclude by May 30th 2030, followed by data cleaning and statistical analyses scheduled for August 1st 2030. The first set of main results is anticipated to be available by the end of 2030. Participants will be enrolled through targeted recruitment based on predefined anthropometric and BMI criteria. The research team includes designated roles for clinical data coordination, biospecimen management, and quality control to ensure standardized data collection and protocol adherence.

### Study participants

The study aims to recruit approximately 3,363 overweight and obese children aged 5–11 years in Shenzhen. Participants will be enrolled through public recruitment campaigns. All eligible children will undergo comprehensive baseline assessments, followed by annual follow-ups over a five-year period. Informed consent will be obtained from guardians at enrollment.

### Inclusion criteria

The inclusion criteria for the study are as follows:

1. Children aged 5–11 years;
2. Resident in Longgang District, Shenzhen;
3. Guardian willing to provide written informed consent;
4. Children classified as overweight or obese according to the Chinese national standard WS/T 586–2018 Screening for overweight and obesity.

### Exclusion criteria

Participants meeting any of the following conditions will be excluded:

1. Refusal to participate or withdrawal of consent at any stage;
2. Missing more than 50% of required baseline measurements;
3. Inability to commit to regular follow-up examinations as scheduled;
4. Planned relocation outside of Shenzhen during the study period;
5. Known diagnosis of congenital cardiovascular anomalies, including congenital heart disease, major coronary artery abnormalities, or other structural cardiovascular malformations;
6. Diagnosis of any severe chronic disease that could independently affect cardiovascular outcomes, such as chronic kidney disease, congenital metabolic disorders, or childhood cancers requiring ongoing systemic therapy.

### Ethics approval and consent to participate

This study was approved by the Research Ethics Committee of the Third People’s Hospital of Longgang, Shenzhen (Approval Number: KY-KT-2025020). Written informed consent will be obtained from all participants’ legal guardians through a digital signature system embedded in a secure WeChat mini-program. Each consent form is electronically signed by the parent or guardian, time-stamped, and automatically stored in the hospital information system (HIS). The system generates a printable record that serves as a legally valid written consent document under Chinese regulations.

### Study procedure

**Fig 1:**
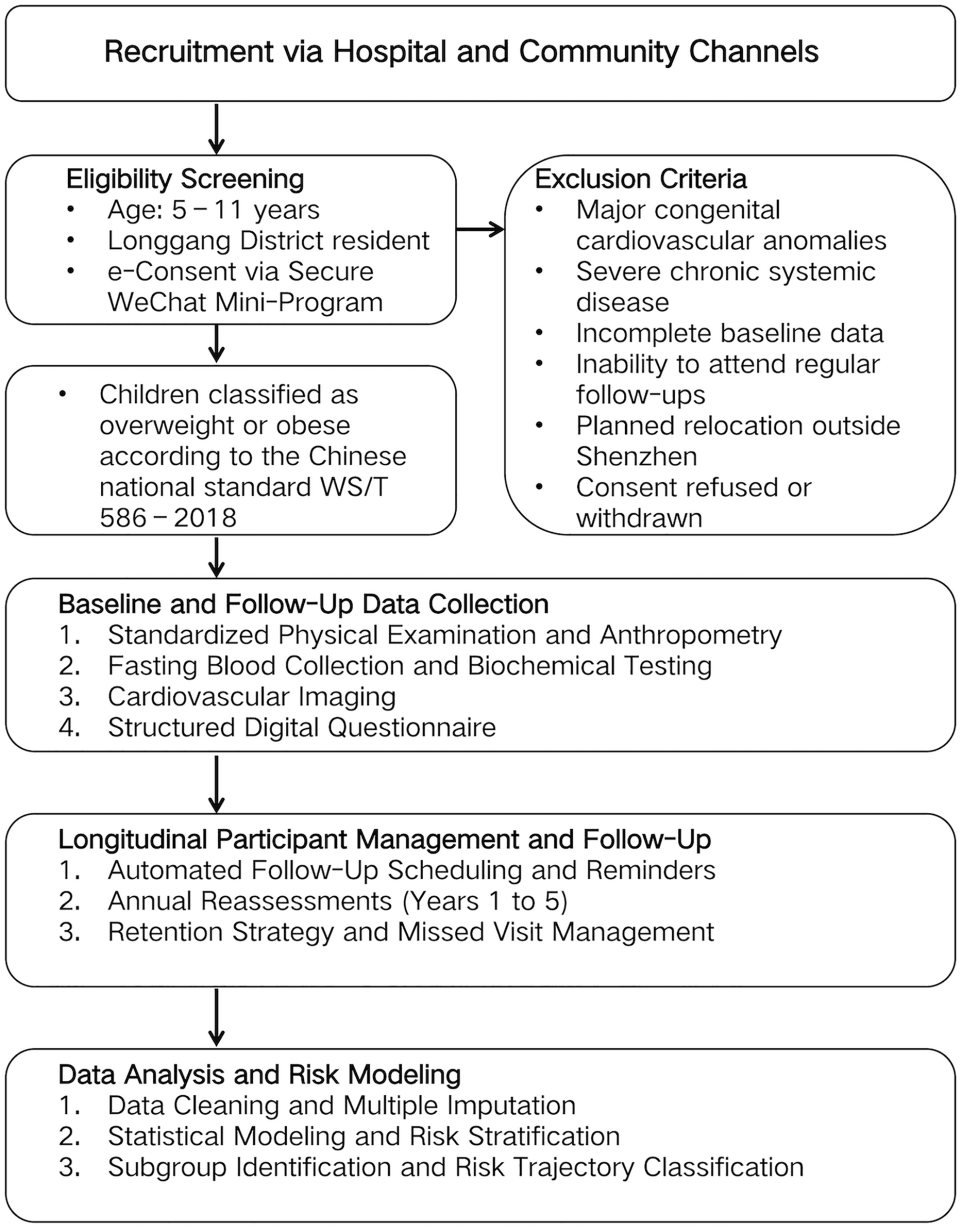
Overview of recruitment, data collection, and follow-up in the SCCS. Children aged 5–11 years were recruited, screened, and enrolled. Multidimensional risk factor data were collected annually and integrated into the HIS, with follow-up and model.

This prospective cohort study will recruit children aged 5–11 years through public enrollment coordinated by the Department of Child Health at the Third People’s Hospital of Longgang. Upon obtaining written informed consent, participants will be registered using their unique Shenzhen Child Healthcare Number. All physical examinations, laboratory tests, and imaging assessments will be conducted at the hospital’s Physical Examination Center, following a standardized protocol to ensure consistency across follow-up visits (Fig 1).

#### Questionnaire-based data collection.

At each baseline visit, guardians complete digital informed consent via electronic signature on a secure WeChat mini-program. They then fill out structured questionnaires covering family health history, lifestyle behaviors, psychosocial factors, and child development. The system includes logic checks and mandatory fields to reduce input errors. Completed responses are encrypted and stored directly in the hospital information system (HIS), allowing linkage with corresponding clinical and laboratory records.

#### Blood sample collection and biochemical measurements.

During each scheduled health examination, fasting venous blood samples are collected by trained nurses at the Physical Examination Center of the Third People’s Hospital of Longgang. Two tubes are drawn after overnight fasting: one EDTA tube for hematological analysis and one plain tube for biochemical and metabolic testing. All samples are processed immediately in the hospital laboratory according to standardized protocols. After initial analysis, aliquots are prepared, labeled with unique identifiers, and stored at −80°C for future assays. Laboratory results are transmitted directly into the HIS. Participants who miss scheduled blood collections are contacted through the research management system and the WeChat mini‑program to arrange resampling within one month.

#### Follow-up scheduling and data management.

A unified research management system, based on the hospital intranet and supported by a WeChat mini-program, is used to coordinate all follow-up procedures. Upon enrollment, each participant is assigned a unique research code and linked to the HIS. The system automatically generates individualized follow-up schedules, with reminders sent to guardians through the mini-program. During each visit, research coordinators assist with identity verification, physical examinations, anthropometric measurements, blood sample collection, and questionnaire completion. All data are entered directly into the research platform. Laboratory and examination results are uploaded to both the HIS and the mini-program, allowing guardians to access their child’s health reports securely. The research platform provides built-in logic checks and data validation functions. Abnormal results are flagged and reviewed by designated research physicians. Missed visits trigger automatic alerts within 48 hours, and coordinators follow up by phone to arrange rescheduling. The system supports flexible appointment options within the hospital and its affiliated units to facilitate participant retention.

We maintain continuous communication with caregivers throughout the follow-up period to document interim health events, including hospitalizations, new clinical diagnoses, acute illnesses, medication changes, or other relevant developments reported between scheduled visits. Because a wide range of unforeseen events may arise during childhood, all reported information is recorded in the study management system and reviewed by the research team. Each event is assessed against predefined exclusion criteria and continuation rules to determine whether it introduces a condition that would substantially interfere with cardiometabolic assessments or compromise the interpretability of longitudinal outcomes. When an event meets these predefined thresholds, withdrawal is considered following a standardized decision pathway.

### Sample size

The sample size for model development was calculated using the pmsampsize package in R, following the approach proposed by Riley [30].This method is specifically designed for clinical prediction models and incorporates key parameters including the number of candidate predictor parameters, anticipated model performance, acceptable overfitting, and outcome prevalence. We specified 20 predictor parameters. Based on a meta-analysis of 134,438 Chinese children and adolescents, the prevalence of dyslipidemia and other metabolic abnormalities was set at 0.19 [31]. The target value for Nagelkerke’s R-squared was set to 0.10, representing modest explanatory power for new models in early childhood populations. To ensure shrinkage of at least 0.90 and stable estimation of all model parameters, the minimum sample size required was 2,803 children, corresponding to 533 expected events and an events-per-parameter (EPP) ratio of 26.6. Accounting for a 20% loss to follow-up, a total enrollment of 3,363 children was planned.

### Data collection and measurements

All measurements and data collection procedures are conducted in the Physical Examination Center of the Third People’s Hospital of Longgang. Standardized protocols are followed, and each data item is linked to an individual-level encrypted profile through the HIS and an integrated WeChat mini-program.

#### Physical examination and developmental assessment.

A full pediatric examination is performed by trained physicians. The evaluation includes inspection and palpation of head and facial features, chest symmetry and thoracic structure, spinal alignment, and limb development. Cardiac auscultation assesses rhythm, intensity, and presence of murmurs, with cardiac boundaries confirmed by percussion. Pulmonary examination includes symmetrical breath sound auscultation. The abdomen is palpated to detect liver and spleen size, masses, or tenderness. Peripheral vascular examination evaluates limb pulses and signs of circulatory compromise. These findings are recorded in structured fields.

#### Ear, nose, and throat evaluation.

Otolaryngological assessment includes external and internal ear inspection (auricle, external auditory canal, tympanic membrane, mastoid region) and hearing screening for both ears. The nasal examination includes assessment of the anterior vestibule, septum, turbinates, and posterior nasal passages. The oropharynx, tonsils, and nasopharynx are inspected for inflammation, asymmetry, or hypertrophy. Smell function is tested with standard odorant strips. Throat and laryngeal structures are examined for gross anomalies. All items are rated as normal or documented with clinical findings.

#### Anthropometric and vital signs.

All anthropometric and physiological measurements are performed under standardized conditions by trained medical staff. Standing height and body weight are measured using a calibrated stadiometer and digital scale, respectively, with subjects wearing light clothing and no shoes. Body mass index (BMI) is calculated as weight in kilograms divided by the square of height in meters. Waist and hip circumferences are measured using a non-stretchable tape; the waist-to-hip ratio is then calculated. Blood pressure is assessed in the seated position after five minutes of rest, using an automatic oscillometric device with appropriately sized cuffs. Body composition is assessed using a segmental multi-frequency bioelectrical impedance analyzer (BIA), which provides quantitative estimates of skeletal muscle mass, intracellular water, extracellular water, total body water, and body fat mass. Additional indices are automatically derived, including protein mass, fat-free mass, fat-free mass index, skeletal muscle mass index, fat mass index, and basal metabolic rate. These results are directly transmitted from the device to the HIS and are available in the integrated research database. All values are stored with corresponding reference ranges and age-specific percentiles to support subsequent risk stratification analyses.

#### Vascular and cardiac examinations.

Cardiovascular function is assessed using standardized imaging protocols. Brachial-ankle pulse wave velocity (baPWV) is measured to assess arterial stiffness. Ankle-brachial index (ABI) is calculated from bilateral systolic blood pressure measurements to detect arterial obstruction. Transthoracic echocardiography is performed by certified sonographers, with evaluation of cardiac chamber dimensions, ejection fraction, wall motion, and valvular function. Fundus photography is performed using a non-mydriatic retinal camera, capturing both optic disc and macula for microvascular analysis.

#### Laboratory tests.

Fasting venous blood samples are collected via a three-tube system: EDTA tube for complete blood count, serum separation tube for biochemical and inflammatory assays, and heparin tube for additional immune assays. Laboratory parameters include: triglycerides, HDL-C, LDL-C, total cholesterol, fasting plasma glucose, fasting insulin, alanine aminotransferase (ALT), aspartate aminotransferase (AST), high-sensitivity C-reactive protein (hsCRP), and interleukin-6 (IL-6). Homeostatic model assessment of insulin resistance (HOMA-IR) is calculated. Samples are processed in the hospital laboratory, with results uploaded to both the HIS and the parent-facing WeChat interface.

#### Questionnaire-based data.

Collection Guardians complete an electronic consent and structured digital questionnaires via a secure WeChat mini-program. The modules cover: family history of chronic diseases, child dietary habits, physical activity levels, screen exposure, sleep patterns, psychological well-being using validated child mental health scales, and detailed socioeconomic information including parental education and household composition. The system enforces logical checks and prevents incomplete submissions. Responses are linked to the child’s health record in real time.

### Study outcomes

The primary outcome of this study is defined as the presence of sustained cardiometabolic abnormalities during the five-year follow-up. At each annual visit, children undergo standardized assessments of lipid profile, glucose regulation, blood pressure, and anthropometric indicators. Each parameter is classified as normal or abnormal based on the child’s age and sex, following thresholds set by Chinese pediatric clinical guidelines, including the Guidelines for the Prevention and Control of Dyslipidemia in Children and Adolescents and the South China Expert Consensus on the Diagnosis and Management of Overweight and Obesity in Children and Adolescents. A child is considered to have developed persistent cardiometabolic risk if two or more indicators are abnormal at two or more time points across follow-up. This outcome captures repeated deviations from age-appropriate health norms and reflects early biological instability. In addition to this binary classification, selected continuous markers such as BMI z-score, waist-to-height ratio, fasting insulin, and blood pressure are used to estimate annualized rates of change based on individual follow-up intervals. These indicators allow for the reconstruction of individual risk patterns and support stratified analyses to identify children who may require early intervention.

### Statistical analysis and modeling

Descriptive statistics will summarize demographic and clinical characteristics. Continuous variables will be presented as mean ± standard deviation or median (interquartile range). Distribution normality assessed by Shapiro-Wilk test. Categorical variables will be expressed as frequencies and percentages. Chi-square tests or Fisher’s exact test will compare categorical variables between groups, while Student’s t-test or Mann-Whitney U test will compare continuous variables based on data distribution.

To characterize longitudinal trends in metabolic and cardiovascular indicators, we will apply linear and generalized linear mixed-effects models. These models incorporate individual-level random intercepts to account for repeated measurements and adjust for age, sex, and baseline values as fixed effects.

To identify groups of children with distinct developmental trajectories, latent class growth analysis (LCGA) will be used. Group assignments will be estimated based on time-varying anthropometric and metabolic indicators. Subsequent models will examine associations between class membership and early-life exposures, including parental health history and household conditions.

Given the multimodal structure of the dataset, including questionnaire responses, blood-based biomarkers, and imaging-derived indicators, integrative modeling will be conducted across domains to support refined risk profiling. In addition to conventional statistical models such as hidden Markov models, Bayesian hierarchical models, and Gaussian graphical models, future analyses will explore representation learning and neural network–based approaches to capture complex, nonlinear relationships across modalities. These techniques are expected to enhance the ability to learn shared features from heterogeneous inputs and improve the sensitivity of trajectory classification.

Missing values will be handled using multiple imputation under missing-at-random assumptions. Model selection will be based on predictive accuracy assessed by repeated k-fold cross-validation. For hypothesis-driven comparisons, multiple testing correction will be applied using Bonferroni procedures.

All statistical analyses will be performed using R software (version 4.0 or later; R Foundation for Statistical Computing, Vienna, Austria). Two-sided p-values less than 0.05 will be considered statistically significant. Results will be reported in accordance with the STROBE (Strengthening the Reporting of Observational Studies in Epidemiology) guidelines for cohort studies.

### Public and patient engagement

This research protocol was developed and will be executed without direct involvement from caregivers or parents. The study design, implementation, data collection, analysis, and dissemination processes were established independently by the research team without consultation from patient representatives or family members.

## Discussion

The long-term consequences of cardiovascular and metabolic risk beginning in childhood are now firmly established in epidemiologic literature [32]. Yet rising rates of pediatric obesity continue to challenge this paradigm, especially in settings where rapid social and environmental transitions outpace the development of monitoring systems. Many children with obesity remain uncharacterized beyond body size, and the lack of longitudinal, multidimensional data limits our ability to distinguish who is at risk, when changes begin, and what signals predict divergence. These gaps are especially pressing in urban regions like Shenzhen, where shifting lifestyles reshape the early-life risk landscape. In this context, the present study offers not only new data, but a structural response to a broader epidemiologic need: to observe, stratify, and eventually intervene before disease becomes entrenched. While landmark cohorts such as the Bogalusa Heart Study [22,23] and the Young Finns Study [24] laid the foundation for linking childhood risk factors with adult cardiovascular outcomes, these studies were conducted in different social, environmental, and technological contexts, limiting their relevance to today’s rapidly urbanizing Chinese settings. In contrast, this study will tries to capture the unique impacts of modern urban environments in China, characterized by rapid lifestyle transitions, dietary changes, and physical activity patterns. Moreover, existing Chinese cohorts, such as BCAMS [28], have provided insights into metabolic risk among youth but have not specifically focused on high-density urban environments. Shenzhen’s rapid economic growth and shifting lifestyle patterns create a unique setting to study how these changes influence early cardiovascular health. In this context, there is a clear need to understand how biological and environmental exposures interact before clinical disease becomes evident. Much of the existing pediatric literature relies on single cross-sectional snapshots, which mask individual variability over time. By contrast, this study collects repeated measures of growth, metabolic function, vascular indicators, and family conditions, enabling the reconstruction of individualized cardiometabolic risk trajectories during a formative developmental stage.

Current pediatric practice continues to rely on BMI as the primary definition of obesity. However, BMI captures only external body size and fails to reflect the internal metabolic or cardiovascular state. Children with similar BMI values may differ markedly in lipid metabolism, insulin sensitivity, systemic inflammation, and vascular function. This mismatch between phenotype and physiology has been observed in both adults and children, often leading to delayed recognition of high-risk individuals. While prior studies have proposed metabolic subtypes of obesity, such classifications typically omit concurrent cardiovascular alterations that may evolve independently or in parallel. Grouping all children above a BMI threshold as biologically equivalent masks important variation and limits the effectiveness of early prevention. To improve precision in pediatric risk prediction, a stratified approach is needed that integrates both metabolic and cardiovascular dimensions within the obese pediatric population.

Several limitations must be acknowledged. Although recruitment focuses on children with local household registration to enhance cohort stability, internal migration may contribute to attrition. Findings may not generalize to rural or less urbanized populations. Some exposure data rely on parental reporting, introducing potential recall bias.

Despite these challenges, this cohort enables a more detailed examination of the early divergence in cardiovascular risk among children with similar body size. Previous studies have described metabolic heterogeneity within childhood obesity, yet most rely on isolated biomarkers or clinical thresholds [33–37]. This study incorporates a broader range of indicators, and these data allow for the identification of distinct developmental patterns that are not captured by BMI alone.

The Shenzhen Children Cohort Study provides a structured framework for advancing pediatric obesity research in China. By following children with obesity through repeated assessments across cardiovascular, metabolic, vascular, behavioral, and family domains, the study can detect early divergence in cardiometabolic risk before clinical symptoms emerge. This longitudinal design enhances comparability across visits and supports models that identify shared developmental pathways and early indicators of elevated risk, rather than relying on single-time-point classifications. Beyond serving as a data platform, SCCS functions as a long-term sentinel for monitoring evolving pediatric health risks in rapidly urbanizing settings. Its modular structure allows new measurements to be added as needed and supports continued follow-up into adolescence and adulthood, forming a long-term system capable of tracking developmental changes with sufficient temporal detail and biological specificity. In the context of rising childhood obesity and fragmented prevention strategies globally, SCCS offers a prototype for multimodal cohort infrastructures that can inform scientific discovery and population-level health planning.
